# Impact of Air Pollution on Health: A Rising Concern in Kathmandu, Nepal

**DOI:** 10.31729/jnma.v63i290.9211

**Published:** 2025-09-01

**Authors:** Bibek Giri, Muhammad Haris, Sameen Mukhtar, Urusha Maharjan

**Affiliations:** 1 Research Institute for Collaborative Development, Kathmandu, Nepal; 2 Department of Medicine, Dow University of Health Sciences, Karachi, Pakistan; 3 Department of Biotechnology, Inland Norway University of Applied Sciences, Norway

**Keywords:** *air pollution*, *burden of disease*, *public health*, *Nepal*

## Abstract

Air pollution, often termed a silent danger, contributes to various chronic and infectious diseases. Despite global improvements, Nepal Still faces significant health consequences. Air pollution has a high impact on the health of Nepalese, especially among vulnerable populations such as young children, the elderly, those with respiratory issues, and pregnant women due to a combination of human activities and geographical factors. The increase in vehicular air pollution in Kathmandu Valley can increase PM2.5 production which can cause respiratory and pulmonary diseases. Strategies to mitigate burden of air pollution in Kathmandu include multi-sectoral approach from the Nepalese and private and public sectors especially through community awareness, strong political will and research implementation strategy. This article aimed to examine present initiatives, point out weaknesses in exiting tactics, and suggest comprehensive methods to lessen the negative effects of air pollution on the socioeconomic well-being and public health of the Kathmandu Valley.

## INTRODUCTION

The Kathmandu Valley, once celebrated for its rich cultural heritage, now faces severe environmental degradation due to rapid urbanization and rising vehicular emissions. Air pollution, particularly hazardous PM2.5 levels, has reached alarming heights exceeding WHO standards by 4.9 times with Kathmandu even ranked as the world’s most polluted city in April 2025.^1,2^ This crisis poses dire health risks, contributing to 7.6% of global deaths in 2015 and projected to cause 24,000 annual premature deaths in Nepal by 2030.^3,4^ Despite WHO guidelines, Nepal lacks a systematic response, exacerbating public health and economic strains.^5^ This article examines current mitigation strategies, identifies gaps, and proposes integrated solutions to address pollution’s socioeconomic and health impacts in the valley.

## CURRENT SITUATION OF AIR POLLUTION IN KATHMANDU AND URBANIZATION

In 2016, Nepal was ranked 177^th^ out of 180 countries for air quality in the Environmental Performance Index (EPI), reflecting severe pollution levels.^6^ Kathmandu is among the most polluted cities in Asia, with urban areas recording a peak fine particulate matter (PM2.5) concentration of 140 μg/m^3^ in 2017—ten times higher than the WHO guideline.^7^ By 2019, the national PM2.5 average was 44.46 μg/m^3^, classified as ‘unhealthy for sensitive groups,’ posing risks to children, the elderly, individuals with respiratory conditions, and pregnant women.^8^ That year, Nepal ranked as the 8th most polluted country globally, with Kathmandu’s annual average PM>.s reaching 48 μg/m^3^.^8,9^ Notably, in January 2019, levels spiked to 102.7 μg/m^3^, far exceeding the ‘unhealthy’ threshold. Despite stuctuations—rising from 45.9 μg/m^3^ (2017) to 54.4 μg/m^3^ (2018) before a marginal decline in 2019—no consistent improvement trend is evident.^9^

Kathmandu’s pollution crisis arises from rapid urbanization, projected to expand the urban population to 60 million by 2040, up from 4.6 million in 2014.^10,11^ This growth has escalated private vehicle use due to inadequate public transit, while poor waste management practices, including open burning, further degrade air quality.^9^ Weak enforcement and insufficient disposal infrastructure exacerbate the issue, particularly during winter when wood and coal combustion for heating intensifies.^9^ Geographic constraints compound the problem; the valley’s mountainous terrain traps pollutants, with factors like wind patterns and meteorology influencing the accumulation of PM2.5, PM10, and US AQI levels.^12^

## CHALLENGES AND HEALTH IMPACTS OF AIR POLLUTION

Exposure to air pollution, both short- and long-term, is linked to numerous health conditions, including chronic obstructive pulmonary disease (COPD), lung cancer, asthma, respiratory infections, and pulmonary insufficiency.^13^ The respiratory system is particularly vulnerable, as inhaled heavy metal pollutants can damage lung cells and impair function.^14^ Fine particulate matter (PM2.5), classified as carcinogenic by the International Agency for Research on Cancer, penetrates deep into the lungs and is a major contributor to cancer risk.^15^ In Kathmandu Valley, rising vehicular emissions exacerbate PM2.5 levels, heightening the risk of respiratory and pulmonary illnesses. Research indicates a strong correlation between elevated air quality index values and asthma exacerbations, alongside reduced pulmonary function in non-viral infections.^16^ Vehicular emissions in the valley were estimated to produce 9,646.40 tons of PM2.5 annually, with studies demonstrating that a 10 g/m^3^ increase in PM2.5 raises cardiovascular and lung cancer mortality risks.^17,18^ Furthermore, PM10 pollution in Kathmandu has been associated with approximately 171,322 premature deaths per year, while PM2.5-induced lung cancer mortality is highest in Lalitpur district, and all-cause mortality peaks in Kathmandu district, underscoring the disproportionate burden in densely populated, high-emission zones.^19,20^ Similar trends are observed in Delhi, India, where rising pollution levels correlate with increased allcause mortality and morbidity.^21^

Children in urban areas face heightened asthma risks due to air pollution, with ozone and PM2.5 exposure linked to airway changes.^16,22^ An estimated 237,000 annual child deaths result from respiratory infections caused by ambient pollution.^23^ Due to developing immune systems and higher air intake relative to body size, children and adolescents are more susceptible to pollution-related harm.^15^ Prenatal exposure also affects fetal growth, birth weight, and lung development, with maternal PM2.5 exposure contributing to ~18% of global preterm births.^24-27^ Globally, air pollution accounts for 122 million years of life lost (YLLs), with 10% occurring in Africa and 8% in Asia due to childhood lower respiratory infections (AAP-LRIs). While Asia reports the highest mortality rates, Africa bears the greatest per capita YLLs. Between 2010 and 2015, childhood AAP-LRI deaths in Asia declined by nearly 30%, yet sub-Saharan African children face a five-year reduction in life expectancy due to pollution.^23^

Elderly populations are also at risk, particularly in regions with high NO2 and O3 concentrations, such as eastern and central China, where pollution increases health shocks among those aged 60-69.^28,29^ Poor air quality discourages physical activity in older adults, exacerbating risks of cardiovascular disease, obesity, and diabetes.^29^ Additionally, traffic police in urban centers like Kathmandu endure prolonged exposure to vehicular dust, leading to eye and skin ailments.^30^ Despite growing evidence, research on air pollution’s health effects in Nepal remains limited. Given the inevitable consequences of worsening air quality, further studies are essential to monitor impacts, raise public awareness, and prompt regulatory action in Kathmandu.

## STRATEGIES AND RECOMMENDATIONS TO ADDRESS AIR POLLUTION

Nepal, akin to many regions in South Asia, is undergoing swift urbanization, notably within the Kathmandu Valley. Rapid urbanization has come with increased demand for motorized travel, rising CO_2_ emissions, and substandard urban air quality, characterized by particulate matter (PM) concentrations more than 6.5 times the World Health Organization’s (WHO) air quality guidelines, landing Kathmandu as the most polluted cities in the world, posing a serious health risk for thousands of Nepalese.^2,31,32^ In addition to constituting 23% of global energy-related greenhouse gas emissions, fossil fuel consumption in motorized transportation emits exhaust fumes containing PM, including black carbon, which is hazardous to human health and a contributor to climate change.^33^ The United Nations Environment Programme estimates that annually, 2.4 million premature deaths resulting from outdoor air pollution could be prevented, and mitigating short-lived climate pollutants promptly could reduce global warming by up to 0.5°C by 2050.^34^

Thus, for cleaner and healthier air in Kathmandu, the Urban Health Initiative (UHI) pilot program was held in Kathmandu Valley, for the first time in Asia in 2019.^1^ It is imperative to implement multifaceted strategies and recommendations to ensure a healthier and sustainable environment for all. Shortterm interventions include the provision of masks and air purifiers, offering immediate relief from harmful pollutants, and safeguarding public health. Open fires and the burning of solid waste must be prohibited in the Kathmandu Valley, alongside implementing effective waste disposal practices. Hazardous waste should undergo high-temperature incineration or treatment to ensure the safe disposal of residues.^35^ Long-term interventions include investing in green parks, corridors for shading and cooling, and pedestrian zones within the city which not only enhances the aesthetic appeal but also contributes to reducing air pollution by increasing green cover and reducing vehicular congestion.^1^

Educating the public about the adverse effects of air pollution and engaging communities in initiatives like tree planting drives can foster a collective effort towards cleaner air. Knowledge and awareness regarding the health risks associated with poor air quality remain insufficient, leading to a lack of basic precautionary measures among the populace.^36^ Advocacy for policies that impose stricter regulations on driving within the inner core of the city, particularly in heritage areas, coupled with the expansion of sidewalks and bike lanes to enhance public accessibility and safety. Embracing clean energy sources like solar and wind power, along with promoting sustainable modes of transportation such as electric vehicles, cycling, and using public transport instead of private vehicles can significantly reduce emissions. Transitioning to low-carbon transport is pivotal in combating air pollution, given that transportation contributes 27% and 22% of total global PM2.5 (less than 2.5 micrometers in diameter) and PM10 (less than 10 micrometers in diameter) emissions, respectively.^37^ Low Sulphur fuels should be used as they reduce the emissions of ultra-fine sulphates which are a serious threat to health.^6^

In 2021, Nepal adopted the Green, Resilient and Inclusive Development (GRID) approach to systematically tackle the repercussions of COVID-19 and the nation’s structural hurdles, a high vulnerability to climate change and environmental degradation, and large infrastructure gaps. The country is currently working on developing the GRID Strategic Action Plan, and air pollution is a top priority in this action plan.^3^ Additional contributors to air pollution in Nepal include the utilization of wood, dung, and agricultural waste for residential cooking and urban transportation. Addressing these issues entails embracing cleaner technologies and practices. The World Bank is actively supporting the adoption of cleaner technologies, including electric vehicles and cookstoves, as part of the inaugural GRID Development Policy Credit operation.^3^ Clean air represents a quintessential case of regional public goods, necessitating collective action among countries, cities, and municipalities sharing the same airshed due to transboundary airflows. Nepal is not isolated in its struggle against air pollution, some of Kathmandu’s pollution originates from neighboring countries, while Nepal’s emissions affect downstream areas. Both regional and global cooperation is critical to curbing air pollution.^3^ Therefore, stronger regulatory policies and effective collaboration between government bodies, non-governmental organizations (NGOs), and international agencies are essential to enforce environmental standards and implement comprehensive solutions.

Although the adverse health consequences of air pollution in Kathmandu are well-documented, the formulation and execution of effective mitigation strategies remain hindered by the various barriers. Foremost among these is the absence of sustained political commitment, which manifests in fragmented policymaking across multiple governmental bodies, inadequate enforcement of existing environmental regulations, and a tendency to prioritize expedient, short-term measures over comprehensive, long-term reforms that may be politically contentious. These governance limitations are further exacerbated by fiscal constraints, as Nepal’s restricted financial capacity impedes investment in critical infrastructure, such as modernized public transportation systems and advanced regulatory technologies, necessary for substantive air quality improvement. Compounding these institutional and economic barriers is a pronounced socio-behavioral resistance, illustrated by public opposition to vehicle usage restrictions. This resistance reflects a broader disconnect between policy initiatives and public buy-in, driven by limited access to viable alternatives and insufficient public awareness regarding the full spectrum of health risks associated with air pollution. Additionally, the transboundary nature of pollution originating from the Indo-Gangetic Plain introduces a layer of diplomatic complexity, as regional cooperation is frequently subordinated to overarching geopolitical considerations. Therefore, acknowledging these multifaceted barriers spanning political, financial, social, and regional dimensions is essential for designing realistic and resilient interventions to address Kathmandu’s air quality crisis.

## CONCLUSION

Air pollution has various health effects on the general population including the residents of Kathmandu. The current situation of this issue in Kathmandu is linked to a combination of human activities and geographical factors posing different health problems including respiratory diseases, cardiac diseases, cancers, etc. Strategies to curb this menace include a multisectoral approach from the Nepalese people, the private and public sectors especially through community awareness, strong political will and research implementation strategy.
